# CCR5 on the NK Cells and its Ligand (RANTES) Expressions are Disrupted in South-Eastern Iranian Patients With Chronic Hepatitis B Infection

**DOI:** 10.5812/ircmj.12458

**Published:** 2014-04-05

**Authors:** Vahid Mirzaee, Jahanbano Shahriari, Masomeh Hajghani

**Affiliations:** 1 Department of Internal Medicine, Faculty of Medicine, Rafsanjan University of Medical Sciences, Rafsanjan, IR Iran; 2 Hematology Laboratory, Bahonar Hospital, Kerman University of Medical Sciences, Kerman, IR Iran

**Keywords:** Chronic HBV Infection, CCR5, RANTES, NK Cells

## Abstract

**Background:**

CCR5 is a receptor for CCL3 (MIP-1 α), CCL4 (MIP-1 α) and CCL5 (regulated on activation normal T cell expressed and secreted (RANTES)) and play important roles in recruitment of NK cells to the HBV infected liver.

**Objectives:**

The main purpose of this study was to investigate the expression levels of CCR5 on the NK cells and also serum levels of RANTES in chronic HBV infected (CHI) patients.

**Materials and Methods:**

In this descriptive study 63 CHI patients and 96 healthy controls were evaluated regarding CCR5 expression on the NK cells and serum levels of RANTES using flow cytometry and ELISA techniques, respectively. Real-Time PCR technique also was used for HBV-DNA quantification.

**Results:**

The results revealed that CCR5 expressing NK cells and serum levels of RANTES were decreased significantly in the CHI patients in compare to healthy control.

**Conclusions:**

Based on the results it can be concluded that NK cells of Iranian CHI patients are unable to express adequate levels of CCR5 and expression levels of RANTES by immune cells also are defected in CHI patients, hence, the migration of NK cells to the infected hepatocytes and HBV eradication from the cells is interrupted.

## 1. Background

The prevalent form of the hepatitis B in Iran is chronic hepatitis B infection (CHI) and could be one of the main causes of cirrhosis and hepatocarcinoma (1). The main features of CHI are persistent hepatitis B infection in which HBV is not eradicated completely from hepatocytes (2). The reasons that lead to stability of HBV infection in CHI are not cleared yet but researchers believe that genetical and epigenetical differences between CHI patients and clearance group may be responsible for various expressions of immune related molecules (3). Chemokines, CCL3 (MIP-1), CCL4 (MIP-1) and especially CCL5 (regulated on activation normal T cell expressed and secreted (RANTES)), are important molecules for recruitment of activated immune cells to the HBV infected liver (4). The specific receptor on the immune cells for CCL5 is CC receptor 5 (CCR5) that is expressed on the T lymphocytes, macrophages and natural killer (NK) cells (4, 5). Previous studies revealed that interaction of the chemokines with CCR5 lead to activation of Th1 lymphocytes to response against viral infections (6, 7). CCR5 also play important roles in migration of immune cells to the inflamed liver (8). Therefore, mal-expression of the receptor may lead to failure immune response against viral hepatitis. Interestingly, we have previously shown that the expression of CCR5 was decreased on the NK cells (4) and T cytotoxic lymphocytes (5) of occult HBV infected (OBI) patients. Therefore, it seems that CCR5 can play important roles in immune responses against HBV infection in CHI patients. The main innate immune cells that are involved in immunity against viral infections including hepatitis B are NK cells (9). Previous studies have shown that CCR5 is a main factor for migration of NK cells to the infected liver (10).

## 2. Objectives

Hence, based on the above introductory comments, we have investigated the expression levels of CCR5 on the NK cells and also serum levels of its ligand (CCL5) in the South-East Iranian CHI patients.

## 3. Material and Methods

### 3.1. Subjects

Peripheral blood samples (with and without anti-coagulant) were collected from 96 healthy controls and 63 CHI patients from Yazd and Rafsanjan, central and south-eastern regions of Iran, respectively. HCV or HIV co-infection and under treatment with immune-modulator drugs were considered as external criteria for the patients. CHI patients were selected according to the “Guide of Prevention and Treatment in Viral Hepatitis” (11). Criteria controls were selected with the same age, sex and socio-economical status. Separation of the serums was done during 8 hours after blood collections and the serum samples were stored at -20 ºC for future analysis, while, the samples with anti-coagulant were used for DNA extraction. Written informed consent was obtained from all of participants prior to sample collection. This study was approved by the Ethical Committee of the Rafsanjan University of Medical Sciences.

### 3.2. HBV Serological Markers Detection

All of samples were tested regarding HBsAg and HBeAg using ELISA kits (Behring, Germany) according to the manufacture guidelines.

### 3.3. HBV-DNA Extraction and Real-time PCR Condition

Viral DNA was purified from 200 μL of HBsAg positive serums using a commercial kit (Cinnaclon, Iran) according to manufacture guidelines. HBV-DNA copy numbers also were evaluated using a commercial kit from Primer Design Company (UK) following the manufacturer’s instructions.

### 3.4. Chemokine Assay

RANTES serum levels were detected using an ELISA kit (eBioscience, ESP) in CHI and healthy control groups immediately after blood collection according to the manufacturer’s guidelines. The sensitivity of the kit was 2 pg/ml and inter- and intra-assay assessments of reliability of the kit were conducted (CV < 14% and CV < 0.03%, respectively).

### 3.5. Monoclonal Antibodies

The fluorescent monoclonal antibodies (mAb) that were used in the study were as follows:

1. Mouse anti-human CD195 conjugated with FITC (fluorescein isothiocyanate) (clone: 2D7/CCR5, isotype: mouse IgG2a, ) (BD, USA) and mouse antibody conjugated with FITC (IgG2a, , clone; G155-178) (BD, USA) as its isotype-matched negative control.
2. Mouse anti-human CD8 conjugated with PE (phycoerythrin) (clone: RPA-T8, isotype: mouse IgG1, ) (BD, USA) and mouse antibody conjugated with PE (IgG1, clone; MOPC-21) (BD, USA) as its isotype-matched negative control.

### 3.6. Flow Cytometry Analysis

In order to detection of CCR5 on NK cells in CHI patients and healthy controls, peripheral blood samples were incubated with mentioned monoclonal antibodies and their isotype-matched negative controls according to the manufacturer’s instructions. Briefly, red blood cells lysis was done using RBC lysis solution (BD, USA) and peripheral blood mononuclear cells (PBMCs) were washed 3 times by PBS. PBMCs were treated with 20μl of PE conjugated anti-CD8 and FITC conjugated anti-CD195 and then cells washed after 30 minutes incubation. 1 10^4^ cells were analyzed by Partec system model PAS. Intensity of CCR5 on NK cells also were obtained by provided software in Partec system model PAS.

### 3.7. Liver Function Tests (LFTs)

Serum levels of alkaline phosphatase (ALP), alanine aminotransferase (ALT), aspartate aminotransferase (AST), direct bilirobin (DB) and total bilirobin (TB) was evaluated using a commercial kit (MAN Ltd Company, Iran).

### 3.8. Statistical Methods

Results were analyzed by T-test and Anova statistical methods. The P value of less than 0.05 considered significant.

## 4. Results

**Figure 1.**
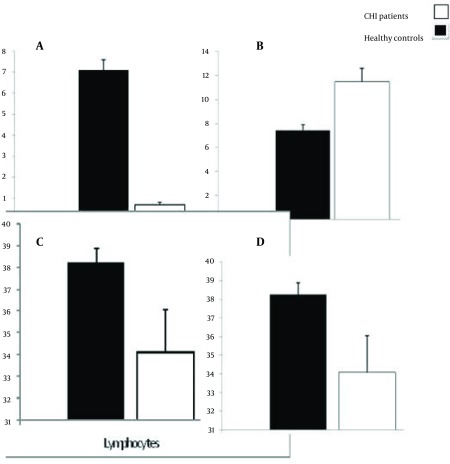
Illustrates the Precent of CCR5+ NK Cells A) BK Cells B) and Total lymphocytes C) and Also Intensity of CCR5 on the NK Cells D) of Data are Shown as Mean SD

**Figure 2.**
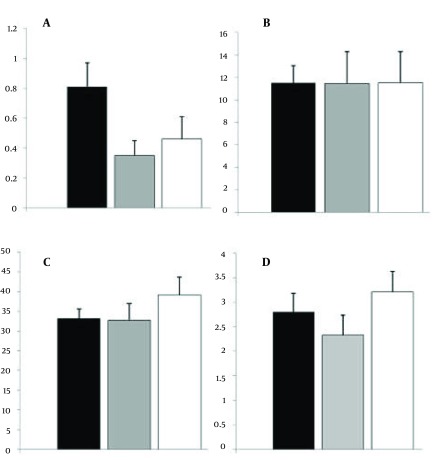
Illustrates the Percent of CCR5+ NK Cells A) Total NK Cells B) and Total Lymphocytes C) and Also Intensity of CCR5 on the CCR5+ NK Cells D) in Groups Carried out Fewer Than 20000 (Black Column) Between 20000 to 400000 (Gray Column) and Upper Than 1000000 (White Column) HBV-DNA Copy Number

**Figure 3.**
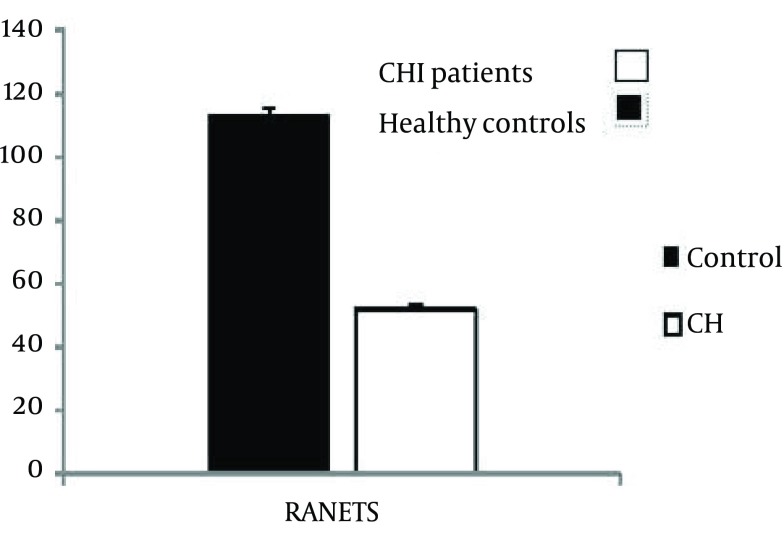
Illustrates the Serum Levels of RANTES in the CHI Petients and Healthy Controls. Serum Levels of RANTES Were Significantly Decreased in the CHI Patients in Compare to Healthy Control

Our results demonstrated that the percent of CCR5 +/NK cells in the CHI patients was 0.7 0.12 while, it was 7.1 0.5 in the control group. Statistical analysis revealed that the difference is significant (P < 0.001) (Figure 1). The results also showed that the intensity of CCR5 was significantly decreased on the CCR5+/NK cells (P < 0.001) (Figure 1). Although, the percent of whole lymphocytes were decreased in the patients in compare to controls (p< 0.001), but, the percent of NK cells were increased (Figure 1). The results of our study also demonstrated that all of the CHI patients were positive for HBsAg with detectable HBV-DNA and five (8.3%) of the CHI patients were HBeAg positive with high HBV-DNA copy numbers (more than 1000000 CN/ml). HBV-DNA quantification showed that 40, 12 and 11 of patients carried out fewer than 20000, between 20000 to 400000 and upper than 1000000 HBV copy number/ml. Statistical analysis revealed that there were no differences between the various HBV-DNA copy number groups regarding percent of CCR5+/NK cells, intensity of CCR5 on the NK cells and also percent of lymphocytes (Figure 2). Serum levels of AST, ALT, ALP, DB and TB were normal in all of the CHI patients and healthy controls with non-significant differences (P > 0.1) (Table 1). Our results also demonstrated that serum levels of RANTES were 51.9 1.66 and 113.5 1.91 in CHI patients and healthy controls, respectively (Figure 3). Statistical analysis revealed that the difference is significant (P < 0.001).

**Table 1.** Demographic and Laboratory Information of CHI Patients and Healthy Controls

Factor	Healthy controls	CHI patients
**Age**	38.41 ± 7	35 ± 9
**Sex**		
Male	31 (48.3%)	28 (46.6%)
Female	29 (51.7%)	32 (53.4%)
**HBsAg** ** positive**	0	All of them
**HBeAg** ** positive**	0	5 (8.3%) of them
**Liver function tests (LFT)**		
ALT	28 ± 9	27 ± 12
AST	29 ± 5	28 ± 11
ALP	240 ± 20	270 ± 40
DB	0.1± 0.08	0.2 ± 0.1
TB	0.6 ± 0.1	0.7 ± 0.2

## 5. Discussion

CCL5 is an important chemokine during immune responses against viral infections (12-14). Previous studies showed that CCR5 is the specific receptor for the chemokine that is expressed by several immune cells including CD8 positive T cells and NK cells (4, 5). Therefore, any alteration in CCR5 and CCL5 expression can lead to imperfect immune responses against viral infection, especially HBV infection (12, 15). Our results demonstrated that the percent of NK cells was significantly increased, while, CCR5+/NK cells and also its intensity was decreased on the CCR5+/NK cells of the CHI patients. Although, increased number of NK cells can be a good immune responses against HBV infection but down regulation of CCR5 and also CCL5 in the CHI patients may lead to block of migration of NK cells to the infected hepatocytes to eradicate HBV. Additionally, the results showed that the percent of total lymphocytes were also declined in the CHI patients in compare to healthy controls. Therefore, it seems that CHI patients are unable to produce enough umber of lymphocytes. Based on our results it can be concluded that Iranian CHI patients not only are defected in producing enough number of total lymphocytes but also are unable to produce CCR5+/NK cells and CCL5. In agreement with our results, TrehanPati et al. revealed that CHI patients are unable to express suitable amount of CCR5 on the CD4+ T cells (16). We also previously showed that the CD8 positive T cells (5) and NK cells (4) of OBI patients were also defected in CCR5 expressions. Thus, it seems that decline CCR5 expressions are associated with the persistence of HBV (chronic and occult form). Based on reported by several researchers it seems that CCR5 and CCL5 are also genetically associated with CHI because several studies showed that genetical variations of CCR5 gene are associated with CHI (2, 14, 17-19). It is unclear yet that why CCR5 and CCL5 are down-regulated in CHI patients but genetical parameters such as CCR5 D 32 mutation and polymorphisms within CCR5 and CCL5 genes as well as epigenetical parameters may affect their expression (14, 20, 21). Therefore, based on the fact that Iranian populations are vary in the ethnic and genetical background, hence, more studies on the other regions of Iran on the CHI patients are needed to be done.

Finally, CCR5 and CCL5 expressions are defected in the CHI patients and it possibly lead to disrupted NK cells recruitment to the HBV infected hepatocytes, hence, NK cells may be unable to eradicate HBV from the hepatocytes. Collectively, mal-expression of CCR5 is one of the several imperfect immune responses against HBV and other factors of immune responses of the CHI patients need to be studies.
